# Production and Properties of a Thermostable, pH—Stable Exo-Polygalacturonase Using *Aureobasidium pullulans* Isolated from Saharan Soil of Algeria Grown on Tomato Pomace

**DOI:** 10.3390/foods5040072

**Published:** 2016-10-29

**Authors:** Leila Bennamoun, Serge Hiligsmann, Scheherazad Dakhmouche, Amel Ait-Kaki, Fatima-Zohra Kenza Labbani, Tahar Nouadri, Zahia Meraihi, Benedetta Turchetti, Pietro Buzzini, Philippe Thonart

**Affiliations:** 1 Laboratoire de Génie Microbiologiques et Applications, Faculté des Sciences de la Nature et de la Vie, Département de Biochimie et Biologie Cellulaire & Moléculaire, Université Frères Mentouri Constantine, Route Ain El Bey 25017, Algeria; scheherazad2002@hotmail.com (S.D.); ait-kaki.amel@umc.edu.dz (A.A.-K.); labkenza@yahoo.fr (F.-Z.K.L.); nouadri2000@yahoo.fr (T.N.); meraihi27@yahoo.com (Z.M.); 2 Walloon Center of Industrial Biology, University of Liège, Boulevard du Rectorat 29, B40 Building, 4000 Liège, Belgium; serge.hiligsmann@ulb.ac.be (S.H.); p.thonart@ulg.ac.be (P.T.); 3 Department of Agricultural, Food and Environmental Science, Industrial Yeasts Collection DBVPG, University of Perugia, Borgo XX Giugno 74, I-06121 Perugia, Italy; benedetta.turchetti@unipg.it (B.T.); pietro.buzzini@unipg.it (P.B.)

**Keywords:** exo-polygalacturonase, *Aureobasidium pullulans*, tomato pomace, response surface methodology, characterization

## Abstract

Polygalacturonase is a valuable biocatalyst for several industrial applications. Production of polygalacturonase using the *Aureobasidium pullulans* stain isolated from Saharan soil of Algeria was investigated. Its capacity to produce polygalacturonase was assessed under submerged culture using tomato pomace as an abundant agro-industrial substrate. Optimization of the medium components, which enhance polygalacturonase activity of the strain *Aureobasidium pullulans*, was achieved with the aid of response surface methodology. The composition of the optimized medium was as follows: tomato pomace 40 g/L, lactose 1.84 g/L, CaCl_2_0.09 g/L and pH 5.16. Practical validation of the optimum medium provided polygalacturonase activity of 22.05 U/mL, which was 5-fold higher than in unoptimized conditions. Batch cultivation in a 20 L bioreactor performed with the optimal nutrients and conditions resulted in a high polygalacturonase content (25.75 U/mL). The enzyme showed stability over a range of temperature (5–90 °C) with an optimum temperature of 60 °C with pH 5.0, exhibiting 100% residual activity after 1h at 60 °C. This enzyme was stable at a broad pH range (5.0–10). The enzyme proved to be an exo-polygalacturonase, releasing galacturonic acid by hydrolysis of polygalacturonic acid. Moreover, the exo-polygalacturonase was able to enhance the clarification of both apple and citrus juice. As a result, an economical polygalacturonase production process was defined and proposed using an industrial food by-product.

## 1. Introduction

The industrial processing of fruits and vegetables generates large quantities of by-products which can be recycled or reused by food, cosmetics, and/or pharmaceutical industries as a potential source of valuable bioactive compounds. Within all vegetables, tomato (*Lycopersicon esculentum*), which is consumed either as a raw fruit or as a processed product, is the second most important vegetable crop in the world and one of the most important components of the Mediterranean diet [1]. 

During tomato processing a huge amount of a by-product is produced: this is known as tomato pomace, which consists of tomato peel and seeds as well as some pulp, representing 10%–40% of total processed tomatoes [2,3]. The management of tomato by-products is considered an important problem faced by tomato processing companies, as they cannot be discharged to the environment. They are used mainly for animal feed or fertilizer [4], as they constitute a promising source of compounds that can be used for their nutritional properties and biological potential. The chemical composition of dried tomato pomace indicates the presence of a significant quantity of fibers, proteins, lipids, carbohydrates (including pectins), amino acids, carotenoids and oligoelements [5,6,7,8]. Several pectin-rich substrates such as wheat [9], deseeded sunflower heads [10], apple pomace [11], grape pomace and orange peels [12,13] have been used for the production of microbial pectinases in both submerged and solid-state fermentation.

Pectinases are pectin degrading enzymes: they have widespread applications in the food industry for clarification of fruit juices and wines. They are used to improve juice yield when pressing and are also employed to regulate the degree of haze and cloudiness. Pectinases are also used during oil extraction and in coffee and tea production where they are used to remove the mucilage coat from coffee beans and to accelerate tea fermentation [14]. Another commercial application includes degumming of plants fibers, paper and textile industry, animal feeds and wastewater treatment due to their ability to degrade pectic polymers [15,16,17].

Several types of pectinases can be found: the most important and widely-used commercial pectinases are endo-polygalacturonase (EC 3.2.1.15) and exo-polygalacturonase (EC 3.2.1.67), which represent around 25% of the global industrial enzyme sales. This figure is expected to increase further by the year 2017 [18,19]. Polygalacturonases (PGs) catalyze the hydrolysis of α-1,4-glycosidic linkages between α-d-galacturonic acid units occurring in pectin scaffold with the introduction of water across the oxygen bridge, by endo- and exo-action [16].

In the last ten years, yeasts have been regarded as an alternative source for the large-scale production of commercial enzymes instead of filamentous fungi, mainly because of their unicellular lifestyle and to their ability to grow in economical culture media. In addition, gene cloning and gene manipulation may improve enzyme production, thus suggesting that commercial enzyme production by yeasts can be considered a well-established certainty [20]. Besides, commercial pectinases of fungal origin are generally a mixture of enzymes such as PG, pectin lyases and pectin methylesterase. On the contrary, yeasts do not secrete pectin methylesterase and their pectinases are mainly PG. Therefore, their pectinases can be used to clarify fruit juice and wine without releasing toxic methanol [19,21].

In the past fifteen years a few studies have reported the ability of yeast-like organisms of the species *Aureobasidium pullulans* to secrete PG [22,23,24,25,26,27]. However, to our knowledge, the use of this species to produce PG from tomato pomace as a basal substrate has not been reported. Furthermore, it is important to discover new pectinase-producing microbial strains and optimize their enzyme production conditions in order to meet increasing demand. 

Optimization of process conditions is one of the most critical stages in the development of an efficient and economic bioprocess. Designing an appropriate production medium and conditions is of crucial importance to improve the efficiency and productivity of microbial synthetic routes. Some statistical designs which help to realize optimal fermentative conditions are available, namely full factorial, fractional factorial or Plackett–Burman design (PBD), and response surface methodology (RSM) [28]. PBD is a method of choice for initial screening of medium components. Further optimization and interaction effects between the components can be studied by RSM. The commonly used response surface designs include central composite design (CCD) [29]. This statistical tool has been used in many biotechnological processes, namely optimization of culture conditions [30], enzyme production [31,32,33,34,35], ethanol production [36], and biomass production [37,38].

Based on the above considerations, the present study was aimed to optimize the production of PG from a strain of *A. pullulans* isolated from (Algerian) Saharan soil in submerged fermentation using tomato pomace as a basal substrate. PBD and RSM were employed for identifying critical variables and optimizing these for maximizing enzyme production. Then, the effect of pH, temperature on the activity of the crude enzymatic extract and mode of action was assessed. A robust PG for bioprocesses involves not only a high catalytic activity but also a stable pH and thermo-stability against different physiochemical conditions. In addition, PG was applied in the citrus and apple juice clarification process.

## 2. Materials and Methods

### 2.1. Isolation and Screening of Yeast Pectinase-Producing Strains

Eight soil samples were collected in December 2010 from palm groves and steppe region from El-M’GHEIR El-Oued province (33°1905900N, 6°5205900E), southeastern Algeria. All were saline, with an electrical conductivity (1/5 at 25 °C) of between 2 and 55 mS/cm. After removing approx. 5 cm of soil from the surface, samples were aseptically collected. To obtain yeasts from soil, 10 g of each sample were added to 90 mL of sterile distilled water, and 100 µL serial dilutions (10^−1^ to 10^−5^) were inoculated onto Yeast Malt (YM) agar plates (glucose 2% malt extract 1%, yeast extract 1%, agar 2%). The plates were incubated at 25 °C for up to 7 days. Yeast colonies grown on Petri dishes were periodically checked; representative colonies of each morphological type were purified, and maintained on YEPG agar slants comprised of yeast extract 1%, glucose 2%, peptone 1%, and agar 2% (Difco, Becton and Dickinson Company, Sparks, MD, USA) stored at 4 °C. The presence of pectinolytic activity was verified on pectin agar medium [39], which consisted of 6.7 g/L Yeast Nitrogen Base (YNB), 10 g/L pectin, and 20 g/L agar (final pH: 7). After cell growth, plates were flooded with a solution of 10 g/L hexaadecyltrimethylammonium bromide. A clear halo around the colony in an otherwise opaque medium indicated degradation of the pectin.

### 2.2. Phenotypic Characterization of Selected Yeast

The yeast strain selected as a result of the previous step was preliminarily characterized using a few conventional phenotypic tests: macroscopic and microscopic morphology, glucose fermentation, carbon (glucose, galactose, sucrose, maltose, trehalose, lactose, raffinose) and nitrogen (nitrate and nitrite) assimilation, growth at different temperatures (10, 20, 25, 30, 35 and 40 °C) and NaCl tolerance at 10%, 15% and 20% [40].

### 2.3. Identification of Selected Yeast

The selected yeast strain, as above reported, was submitted to identification via molecular approach (sequencing of the D1/D2 domain of 26S rRNA gene). DNA extraction was carried out according to Sampaio et al. [41]. DNA was first amplified as a template by the PCR method using the primers V9G (5′-TGCGTTGATTACGTCCCTGC-3′) and RLR3R (5′-GGTCCGTGTTTCAAGAC-3′; Sigma-Aldrich Co). A 600-650 bp region was sequenced by the forward primer (5′-GCATATCAATAAGCGGAGGAAAAG-3′) and the reverse primer NL4 (5′-GGTCCGTGTTTCAAGACGG-3′; Sigma-Aldrich Co). The PCR products were sequenced using commercial sequencing facility (Macrogen, Amsterdam, Netherlands). The sequences obtained were compared with those included in the GenBank database (Blastn freeware from http://www.ncbi.nlm.nih.gov/BLAST) [42]. Phylogenetic analysis was performed on the platform (www.phylogeny.fr) [43]. After identification, the strain was deposited in the Industrial Yeasts Collection DBVPG of the University of Perugia, Italy (http://www.dbvpg.unipg.it) with the number DBVPG 5844 [44]. One copy was also conserved in the Department of Biochemistry and Cellular and Molecular Biology, Mentouri Brothers University (Constantine, Algeria).

### 2.4. Materials

Tomato pomace was obtained from the tomato-processing plant *Maison Latina* (Chelghoum Laid, Algeria). It was sun-dried (25–30 °C, 3–4 days) and ground in a blender. Tomato pomace was then passed through a 0.5 mm opening sieve to obtain fine powder and stored in plastic bags at room temperature until use.

### 2.5. Substrate and Culture Media

The fermentation studies were performed using tomato pomace as a basal substrate. The composition of tomato pomace used in this study (in dry weight basis) was as follows: 20.25% total sugars, 18.31% protein and 5.83% minerals. All experiments were carried out in triplicate and the mean values are reported. The basal medium at 40 g/L of tomato pomace powder was settled at different pH and supplemented with various factors (glucose, lactose, yeast extract, CaCl_2_) according to the experimental design. Fifty mL of production media were distributed in 250 mL Erlenmeyer flasks, autoclaved at 121 °C for 20 min; then cooled to room temperature. An inoculum of 2.5 ×10^7^ cells/mL was used. The Erlenmeyer flasks were incubated at 30 °C for 72 h on a rotary shaker at 150 rpm. The biomass was separated by centrifugation at 5000× *g*, for 15 min at 4 °C. The cell-free supernatant, named raw enzymatic extract (EE), was used as a source of extracellular enzymes. The assay batch cultures were run in triplicate, and mean values were reported.

### 2.6. Polygalcturonase (PG) Activity Assay

PG activity on cell-free supernatants was assayed by the colorimetric method of Miller [45]. The reaction mixture, containing 100 μL of culture filtrate, was incubated with 100 µL of substrate polygalacturonic acid (PGA) from citrus fruits (Sigma-Aldrich, St. Louis, MO, USA) at 1%, *w*/*v*, in 50 mM sodium acetate buffer pH 5 at 40 °C for 20 min. After adding 400 µL of dinitrosalicylic acid (DNS), the mixture was boiled for 15 min. Mixture was finally diluted to 5 mL with distilled water (4.4 mL). The absorbance of the color developed was measured at 540 nm. The enzyme activity was determined from a calibration curve by using galacturonic acid (Sigma-Aldrich) as standard. One unit (U) of PG activity was defined as 1 μmol of galacturonic acid released mL^−1^·min^−1^under the assay conditions.

### 2.7. Experimental Designs

#### 2.7.1. Plackett-Burman Design (PBD)

PBD is a mathematical technique for screening and evaluating the important variables that influence response [46]. It was used in the present study to screen the important variables that significantly influenced PG production. In this study, an 8-run PBD was applied to evaluate five factors (including two dummy variables). Each variable was examined at two levels: −1 for the low level and +1 for the high level. Table 1 illustrates the variables and their corresponding levels used in the experimental design. The PBD and the response value of potentiation of PG activity are shown in Table 2. The effect of the individual variable on potentiation of PG activity was calculated by the following Equation (1):

(1) $$E\left(x_{i}\right)=\frac{2\sum \left(M_{i}^{+}-M_{i}^{-}\right)}{N}$$

where *E* (*x_i_*) is the effect of the tested variable (*x_i_*), *M_i_^+^ and M_i_^-^*are responses (PG activity) of trials at which the variable is at its high or low levels respectively, and N is the total number of trials. Experimental error was estimated by calculating the variance between the two dummy variables using the following Equation (2):

(2) $$V_{eff}=\frac{\sum {\left(E_{d}\right)}^{2}}{n}$$

where, *V_eff_* is the variance of the effect, *E_d_* is the effect for the dummy variable and *n* is the number of dummy variables used in the experiment.

The standard error (SE) of the effect was the square root of *V_eff_* and the significance (*p*-value) of the effect of each variable on PG activity was measured by Student’s *t*-test:

(3) $$t(x_{i})=\frac{E\left(x_{i}\right)}{SE}$$

where, *E* (*x_i_*) is the effect of variable *x_i_*.

The variables whose confidence levels % were ≥90% were considered to significantly affect the potentiation of PG activity.

#### 2.7.2. Central Composite Design (CCD) and Response Surface Methodology (RSM)

The next step in the formulation of the medium was to determine the optimum levels of significant variables for potentiation of PG activity. For this purpose, the RSM, using a CCD (Box and Wilson [47]), was adopted for the augmentation of total potentiation of PG activity. The significant variables utilized were pH, lactose and CaCl_2_. Each one was assessed at five coded levels combining factorial points (−1, +1), axial points (−1.682, +1.682), and central point (0), as shown in Table 3. A total of 17 experiments were conducted. The model was represented by the following quadratic equation:

(4) $$Y=\beta_{0}+\sum \beta_{i}X_{i}+\sum \beta_{\mathrm{ii}}X_{i}^{2}+\sum \beta_{\mathrm{ij}}X_{i}X_{j}$$

where, Y is the predicted response (PG activity); X_i_ and X_j_ are input variables that influence the response Y; β_0_ is the constant term; β_i_ is the *i*th linear coefficient; β_ii_ is the *i*th quadratic coefficient and β_ij_ is the *ij*th interaction coefficient.

Analysis of variance (ANOVA) was conducted to determine the significance of model and regression coefficients. The quality of polynomial equation was judged by determination coefficient (*R*^2^), and its statistical significance was checked by Fisher’s *F*-test. The significance of regression coefficients was tested by Student’s *t*-test. The response surface and contour plots of the model predicted responses were utilized to assess the interactive relationships between the significant variables. Statistical analysis of the data was performed using Minitab software (version 17.1, Minitab Statistical Software, State College, PA, USA). Three-dimensional plots were obtained using the software Statistica version 10.0 (StatSoft Inc., Tulsa, OK, USA). For statistical calculation, the experimental variables *X*_i_ have been coded as *x*_i_ according to the following transformation equation:

(5) $$x_{i}=\frac{X_{i}-X_{0}}{\Delta X_{i}}$$

where *x_i_* is the dimensionless coded value of an independent variable, *X_i_* is the real value of an independent variable; *X*_0_ is the real value of the independent variable at the center point; ∆*X_i_* is the step change value. pH (*X*_1_), lactose (*X*_2_) and CaCl_2_ (*X*_3_) were chosen as three independent variables during the preparation process. Their range and levels were listed in Table 3.

### 2.8. Potentiation of PG Activity in Bioreactor

The verification of the statistical model for potentiation of PG activity was carried out in a 20-L laboratory-scale bioreactor (Biolaffite, France) with a working volume of 15 L. The optimized medium was sterilized in situ at 121 °C for 20 min. Lactose was separately sterilized and added to the medium. A computer connected to the fermenter allowed for the control of fermentation parameters: temperature, agitation, partial pressure of dissolved oxygen and pH. The reactor was aerated using a continuous flow of filtered sterile air of 1 vvm (volume of air per volume of medium per minute). The stirring rate was controlled to provide a minimum dissolved oxygen concentration 60%. The fermentations were conducted at 30 °C. The pH was automatically maintained at 5.16 by addition of 4 N KOH or 4 N H_3_PO_4_. The pre-culture was prepared in a 2 L shake-flask containing 1 L of the same medium used for bioreactor. The flask was inoculated with fresh cells that were grown for 24–48 h on plates. The flask was then incubated on a shaker (150 rpm) at 30 °C. After 24–36 h of incubation, the pre-culture was used to inoculate the bioreactor. Samples were withdrawn and analyzed for potentiation of PG activity.

### 2.9. Influence of Different pH on the Activity and Stability of PG

The optimal pH for enzyme activity was determined by conducting the crude enzyme assay in various pH ranging from 3 to 10 and maintaining the temperature (40 °C) and substrate concentrations (1.0%) constant. For the determination of pH stability, studies were performed by incubating the crude enzyme in various buffers having different pH values for 3 h. The residual activity was determined by performing the enzyme assay. The buffers tested included acetate buffer (50 mM, pH 3.0–5.0), phosphate buffer (50 mM, pH 6–7), Tris/HCl buffer (50 mM, pH 7.5–8.5) and glycine-NaOH buffer (50 mM, pH 9–10).

### 2.10. Effect of Temperature on the Activity and Stability of PG

The optimum temperature for activity was measured by assaying the enzyme at different temperatures ranging from 4 to 90 °C at a constant pH (50 mM acetate, pH 5) and a substrate concentration of 1%. The stability of PG against different temperatures was determined by pre-incubation of crude enzyme without substrate at different temperatures (60, 70, 80, and 90 °C) for 5 h. The samples were taken at 1-h intervals and were assayed for activity.

All the experiments were performed in triplicates and the results mentioned here are the mean values.

### 2.11. Mode of Action of PG

The products of the hydrolysis of polygalacturonic acid by PG were analyzed by thin-layer chromatography. Elution was performed using 1-butanol, acetic acid and water in the proportions 9/4/7 as the mobile phase [48], and the galacturonic acids were detected using 10% sulfuric acid in ethanol. A solution of monogalacturonic acid at 10 g/L was used as a standard. 

### 2.12. Application of Exo-PG in the Clarification of Apple and Citrus Juice

Citrus and apples were purchased from a local market and stored at 4 °C until used. Citrus juice was extracted from citrus using lemon-press device. Apple juice was extracted from 200 g of apple using a lab blender and the extracted pulp was filtered over a muslin cloth to obtain the raw apple juice. Five mL of fresh citrus and apple juice were mixed with 50 U (85 μL) of crude enzyme and incubated at 30 °C for 4 h. Clarifying activity was determined by measuring the reducing sugars released in the supernatant obtained by centrifugation at 5000 rpm for 15 min using the dinitrosalicylic acid (DNS) method [45], weighting the residual pellets, estimating the increase of the clarified juice volume, and color (A_420_).

## 3. Results and Discussion

### 3.1. Identification and Phenotypic Characterization of Strain

Twenty yeasts were isolated from palm groves and steppe region in El-M’GHEIR El-Oued province, southeastern Algeria; among them, one strain exhibited a superior ability in degrading pectin. The results of the phenotypic tests showed that the isolate was able to assimilate glucose, galactose, sucrose, maltose, trehalose, lactose, raffinose, and nitrate and nitrite. Glucose was only weakly fermented. The strain exhibited the ability to grow within 4 and 35 °C, and also tolerated a NaCl concentration of 20%. Based on sequencing of D1/D2 domain of the 26S rRNA sequence and the phylogenetic alignment of homologous D1/D2 sequences, the strain was identified as *A. pullulans* (100% sequence similarity with the Blast sequence FJ150942, belonging to the type strain CBS 584.75). 

### 3.2. Selection of Significant Variables Using Plackett–Burman Design (PBD)

PBD employing eight experiments was used in the first phase of optimization: screening of variables significantly affecting PG activity. The design matrix and the corresponding responses are presented in Table 2. The variables exhibiting significantly (*p* < 0.05) high impact (experimentally-determined) on the synthesis of PG—the analyzed enzyme—were subject to optimization in the consecutive stages of the study. The effect of the factors on potentiation of PG activity is reported in Table 4. A statistically significant (*p* < 0.05) effect on PG synthesis was determined in the case of two variables, i.e., initial pH value of the culture medium and concentration of lactose (Table 4). In addition, the coefficient of determination (*R*^2^) of the model was 0.9908, which explains up to 99.08% variability of the data.

The variation in pH from 5 to 6 was significantly positive in potentiation of PG activity (*p* ≤ 0.027) resulting in an increase of 30.25% (effect of pH = 1.2490 (Table 4)/4.13 PG activity of the basal medium (Table 2) × 100). These results can be explained by the fact that the initial pH may affect both yeast growth and PG activity [49,50,51,52]. PG produced by yeasts and filamentous fungi currently exhibit an acidic optimum pH between 3.3 and 7. Suresh et al. [53] reported that maximum production of pectinase from *Aspergillus niger* and *Aspergillus awamori* was observed at pH 5.

The addition of lactose to tomato basal medium significantly (*p* ≤ 0.008) increased the potentiation of PG activity of 57.39%, thus suggesting that the amount of pectin in tomato pomace and the lactose addition are enough to obtain high levels of PG. Pectin used as the sole source of carbon for PG production initially induced PG activity, and when the medium was supplemented with lactose the production was enhanced. The positive combined effect of pectin with other carbon sources herein observed is in agreement with other studies, for example Mukesh et al. [54], who reported also that the use of cassava waste as substrate in combination with lactose supported maximum pectinase production by *Bacillus* sp. MFW7. 

In contrast to the other analyzed variables, concentration of CaCl_2_ exhibited a significant negative effect (*p* ≤ 0.021) on potentiation of PG activity, leading to a 34.28% decrease. This result is probably due to the excessive amount of calcium used in our study, in close agreement with a recent study that reported that with a concentration of metal ions over a critical value, the enzyme production is low, due to a blockage of secretion of protein into external medium [55]. Calcium plays an important role in the stabilization and the protection of the enzyme from undergoing denaturation [56,57]. Moyo et al. [58] confirmed that Ca^2+^ conferred stability to PG produced by *Kluyveromyces wickerhamii*, isolated from rotting fruits. This may be due to the protective action of calcium chloride against heat inactivation of the pectinase. Similar results have been obtained in the production of pectinase by *Bacillus pumilus* [59]. The effect of various metal ions on pectinase production was studied by several Kashyap et al. [60] who indicated that the addition of either CaCl_2_ or MgSO_4_-7H_2_O to medium resulted in a significant increase (more than three-fold) in pectinase activity. 

Other variables, i.e., glucose, yeast extract (as nitrogen and vitamin source) exhibited only a statistically insignificant variation on potentiation of PG activity, The insignificant influence of yeast extract may be apparently explained by the presence of sufficient amounts of vitamins and proteins in tomato pomace [61] which are necessary for the yeast growth and consequently, for PG synthesis.

Due to their insignificant influence on potentiation of PG by *A. pullulans*, the concentrations of glucose and yeast extract were omitted in the successive stages of optimization. On the contrary, the optimum levels of the three selected variables (pH, lactose and calcium chloride concentrations) were further determined by RSM design.

### 3.3. Optimization of Significant Variables Using Central Composite Design (CCD) and Response Surface Methodology (RSM)

**Figure 1:**
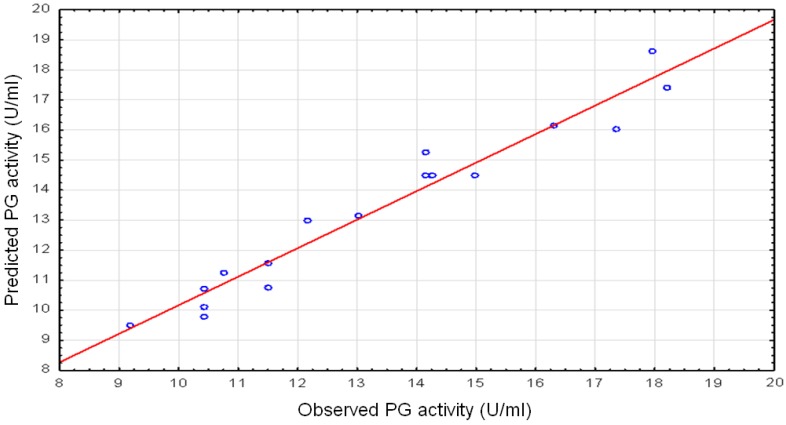
Parity plot showing the distribution of experimental data versus predicted value by the model for potentiation of PG activity.

The variables selected in the previous step were subjected to further optimization. The CCD was used to determine the optimum levels of the three selected variables. Experimental design and results for 17 runs were given in Table 5. The results of the second-order response surface model fitting from ANOVA were presented in Table 6. Table 7 shows the significant coefficients of the full second-order polynomial of PG activity determined by Student’s *t*-test and *p*-values. The empirical relationship between PG activity (Y) and the experimental variables obtained by the application of the RSM is represented mathematically by the following regression equation:

(6) $$Y=14.31-1.40X_{1}+3.94X_{2}+1.69X_{3}+0.05X_{1}^{2}-0.83X_{2}^{2}-2.82X_{3}^{2}-1.78X_{1}X_{2}+0.86X_{1}X_{3}+2.78X_{2}X_{3}$$

where Y is the response for the PG activity and X_1_, X_2_ and X_3_ represent the variables pH, lactose and CaCl_2_, respectively. Furthermore, a correlation was drawn between experimental data and the predicted values by the model as given in Figure 1.

The fitness and adequacy of the model were assessed by the coefficient of determination (*R*^2^). The high *R*^2^ coefficient obtained (0.9421) underlined that the model herein reported is adequate and that only 5.79% of the total variation of potentiation of PG activity is not explained by the model. The predicted *R*^2^ of 0.8558 is in reasonable agreement between the experimental and predicted values for PG activity. The adjusted *R*^2^ corrects the *R*^2^ value for the sample size and for the number of terms in the model. The relatively high-adjusted determination coefficient (*R*^2^_Adj_ = 0.8677) in the present study accounts for a high significance of the model [62].

The ANOVA, through the quadratic regression model, revealed that the second-order response surface model obtained is highly significant. This evidence is also confirmed by the high value of Fisher’s test (F_model_ = mean square regression/mean square residual = 12.66, which is higher than the tabled value F_(9,6)_ = 7.97 for 1% significance level, thus representing a significant model) and the very low probability value (0.001). The *F*-test calculated for regression was significant (*p* < 0.01) at a level of 1%, indicating that the model is appropriate and can adequately explain the variation observed in PG biosynthesis with the designed levels of the selected variables. Theses *p*-values for the model and for lack of fit (0.377), also suggested that the obtained experimental data was accurately fitted by the model.

The Student *t*-distribution and the corresponding *p*-value, along with the parameter estimate are given in Table 7. The estimated parameters and the corresponding *p*-values (Table 7) confirmed that, only the experimental variables (*X_1_*, *X_2_*, *X_3_*), the quadratic term (*X_3_*^2^) and the interaction (*X_2_X_3_*) were significant terms in the empirical relationship between PG activity (*Y*). The positive effects of lactose concentration (*X_2_*) and CaCl_2_ concentration (*X_3_*) indicate that they have a linear effect in increasing potentiation of PG activity, while initial pH (*X*_1_) shows a negative coefficient indicating that it contributes to decrease potentiation of PG activity. The quadratic term of CaCl_2_ concentration (*X*_3_) had also a negative coefficient, meaning that potentiation of PG activity is more related to the linear effect of this variable. However, the interaction (*X_2_X_3_*) contributes to the response at a significant level.

### 3.4. Response Surface Analysis

**Figure 2:**
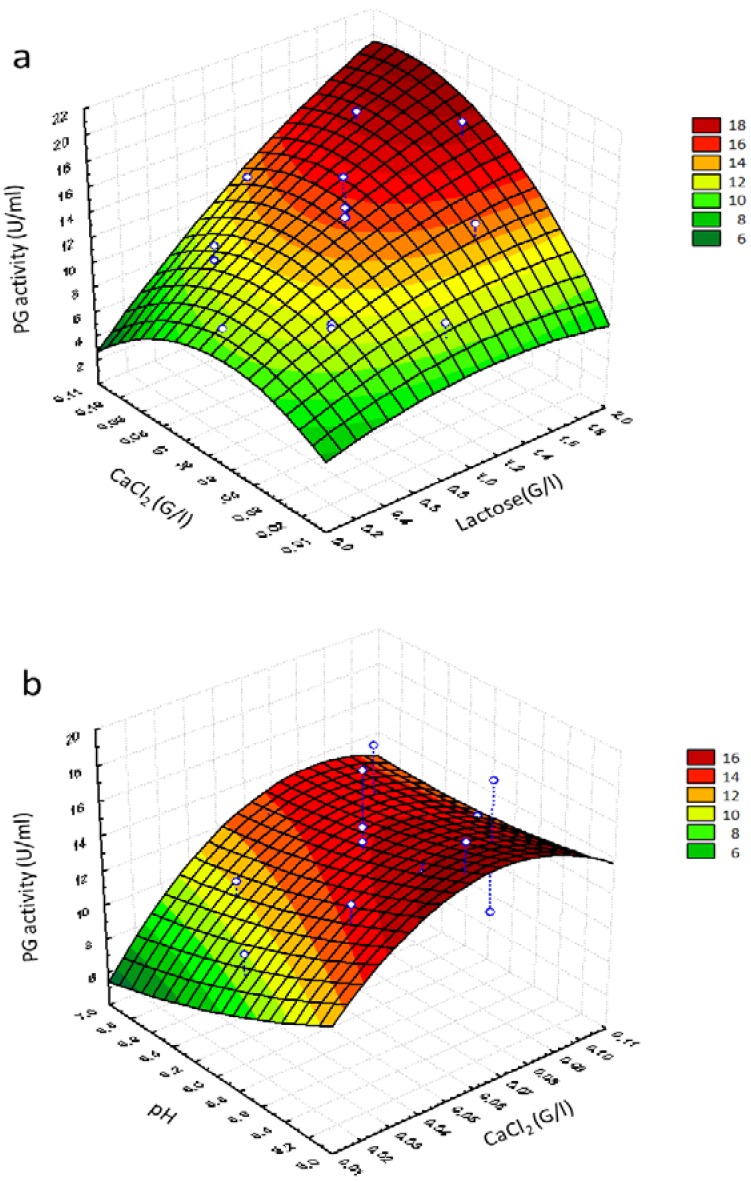

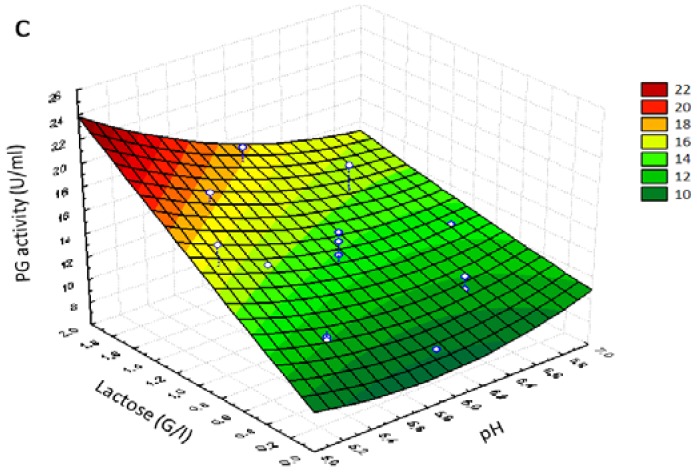
Response surface graphs of potentiation of PG activity by *Aureobasidium pullulans* showing the effect of variables of (**a**) Lactose–CaCl_2_, (**b**) pH–CaCl_2_, and (**c**) pH–Lactose.

The response surface graphs shown in Figure 2 were obtained by the second-order polynomial model. This graph, showing the experimental variation of response (PG activity) when *X_1_*, *X_2_* and *X_3_* (considered two by two) varied within their experimental range (and holding the third factor at fixed center level) was in fact more helpful in interpreting the main effect and interactions.

Figure 2a shows the effect of lactose and CaCl_2_ concentration on potentiation of PG activity and confirmed the existence of an interaction between the two variables (*p* = 0.033) (Table 7). A high potentiation of PG activity was observed when lactose concentration was increased. The maximum production (>18 U/mL) was predicted at the lactose concentration of approximately 1.80 g/L and the CaCl_2_ concentration of approximately 0.09 g/L, thus confirming the calcium stabilizing effect on enzyme production reported by Shanmugaprakash et al. [63].

No significant interactions were observed between pH and CaCl_2_ (Table 7). Low PG values were obtained at about pH 6.2 (Figure 2b), in agreement with the results reported by Suresh et al. [51], who studied the stability of PG synthesized by *Aspergillus awamori* in submerged fermentation as a function of pH, and observed that the enzyme was quickly denatured when pH was raised at values higher than 5.4. Therefore, the maximum of potentiation of PG activity was obtained only at low levels of pH (5.2) and low levels of calcium chloride (approximately 0.09 g/L).

Lactose at higher concentrations and pH at higher values depleted the potentiation of PG activity, while a better combination was higher lactose concentration and lower pH values, which showed a positive effect on potentiation of PG activity (Figure 2c), thus highlighting that these variables act in the opposite manner on the potentiation of PG activity. According to the response surface point prediction analysis, pH 5.16, lactose concentration of 1.84 g/L and CaCl_2_ (0.089 g/L) maximized the yield of PG up to 21.83 U/mL.

### 3.5. Validation of the Quadratic Model

In order to confirm the above mentioned optimized conditions (under the form of a second-order polynomial model reporting the variation of potentiation of PG activity as a function of initial pH, lactose and CaCl_2_ concentration), an experiment for potentiation of PG activity was performed in triplicate. The enzyme production attained (22.05 U/mL) was higher than the CCD predicted PG activity (21.83 U/mL).

**Figure 3:**
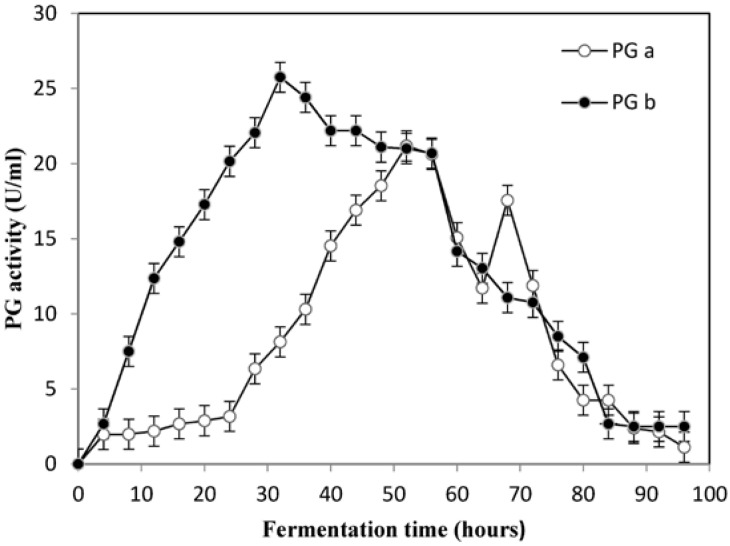
Potentiation of PG activity in shake flasks (**a**) and a laboratory bioreactor (**b**) by *A. pullulans*. The results were presented as mean ± SD, *n* = 3.

Therefore, the optimization procedure allowed increasing PG activity of cell-free supernatants of *A. pullulans* from 4.13 U/mL (Table 2) to 22.05 U/mL. This result is apparently superior to those reported in literature [35,64,65,66]. Thus, the model developed is accurate and reliable for optimizing the potentiation of activity of polygalacturonase by *A. pullulans*. A comparative study of the potentiation of activity of PG was performed on the optimized medium in Erlenmeyer flasks and a 20 L bioreactor. The results revealed higher potentiation of activity of PG in a shorter time when the yeast was cultivated in the 20 L bioreactor. The potentiation of PG activity increased further to 25.75 U/mL in the laboratory bioreactor (Figure 3). The peak in the enzyme activity was attained in 32 h in the bioreactor as compared to 52 h in the shake flasks. A similar reduction in fermentation time was recorded in the production of alkaline pectinase by *Bacillus pumilus dcsrl*, where the optimum production was achieved in 30 h in bioreactor as compared to 40 h in shake flasks [65]. These results confirmed that *A. pullulans* had potential to produce PG in large-scale cultivation. 

### 3.6. pH Stability on the Crude Enzymatic Extracts

**Figure 4:**
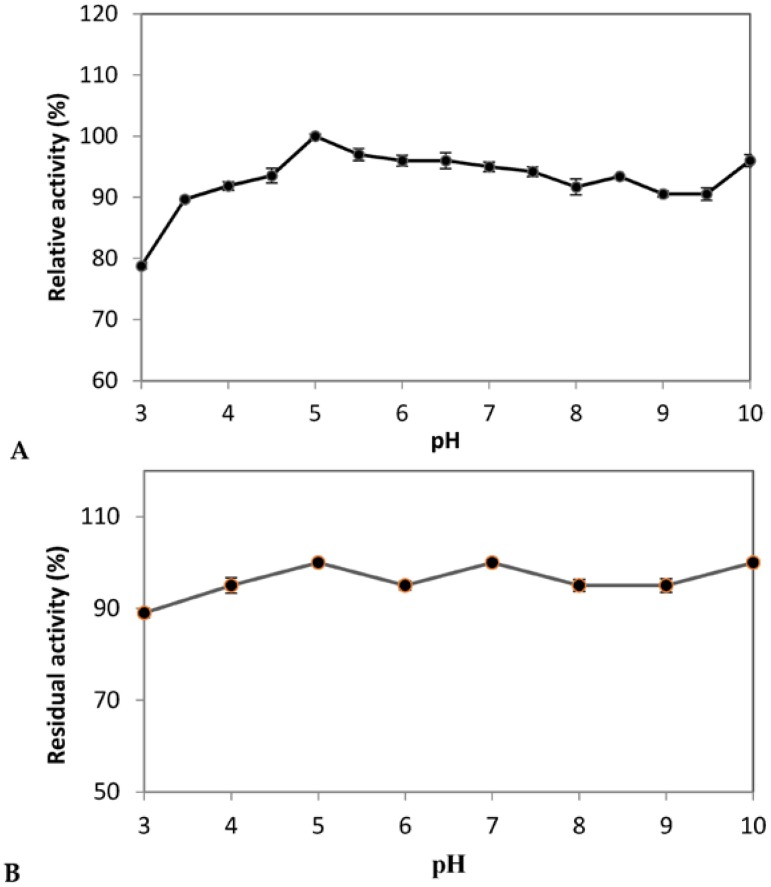
Effect of pH on the activity (**A**) and stability (**B**) of PG of *A. pullulans* isolated strain. Each point represents the mean (*n* = 3) ± standard deviation.

The pH is one of the primary contributing factors that play a crucial role in enzyme activity and stability. Most exo-polygalacturonases currently available in the literature are produced by fungi. These fungal exo-polygalacturonases have maximal activity at a pH range of 4.0–5.0 but lose stability under acidic and basic conditions [16,67]. *A. pullulans* isolated from (Algerian) Saharan soil PG was active over a wide range of pH values and showed more than 90.0% activity at different pH values ranging from 4.0 to 10.0 with the maximum around pH-5.0 (Figure 4A). PG showed a second peak of activity at pH 10.0 with 96% of maximum activity. Probably, there are two isoenzymes. This needs to be confirmed after purification by SDS-PAGE and by zymogram analysis.

The stability of enzyme against various pH is important for its commercialization and was also tested. It was observed that the enzyme is stable over a broad range of pH values and retained its 100% activity at pH 5.0, 7.0 and 10.0 up to 180 min (Figure 4B). Similarly, PG from *Klebsiella sp.Y1* was reported to be stable in a wide range of pH (2–12) in digestive tract of sheep [68]. However, Manachini et al. [69] reported the stability of PG from *A. pullulans* at pH 4.0 to 6.5. 

### 3.7. Thermostability on the Crude Enzymatic Extracts

**Figure 5:**
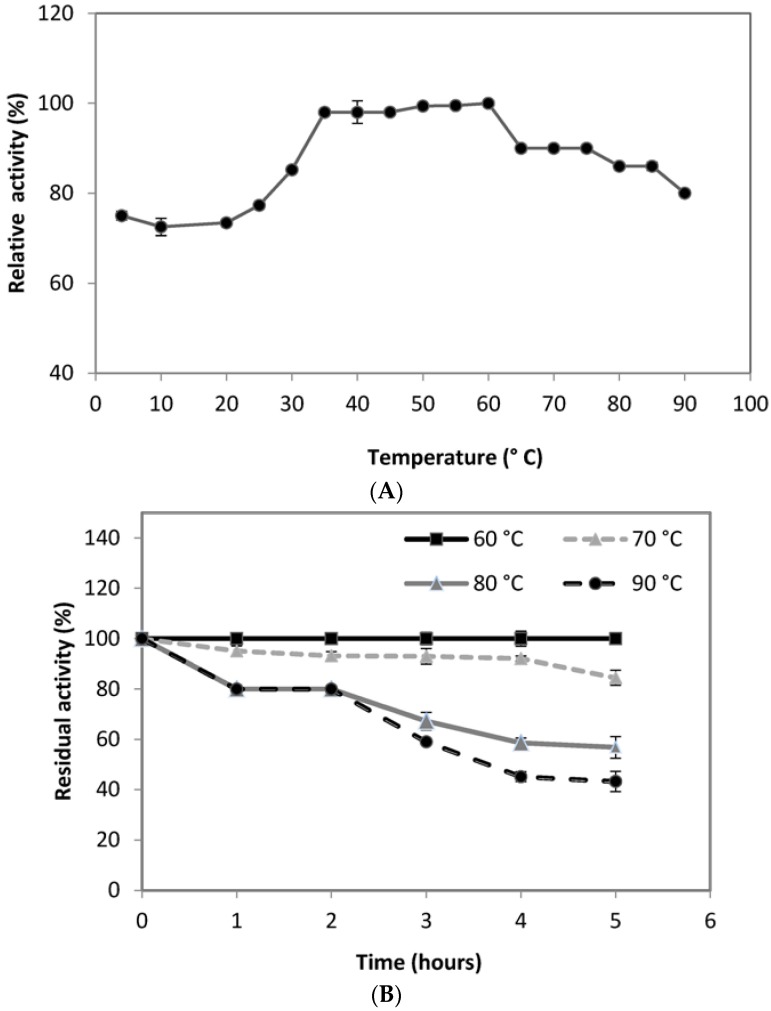
Effect of temperatures on the activity (**A**) and stability (**B**) of PG from *A. pullulans* isolated strain. Each point represents the mean (*n* = 3) ± standard deviation.

Temperature is also significant for the activity and stability of any enzyme. The stability of an enzyme against different temperatures represents the capability of an enzyme to resist against thermal denaturation in the absence of substrate and it is one of the major requirements for the commercialization of the enzyme in industries. It was observed that the PG was active over the broad range of temperature range 4–90 °C with optimum temperature of 60 °C (Figure 5A), slightly higher than that reported from *Aureobasidium pulluans LV10* [69]. When maintained at 60 °C for 1 h, the retained activity of PG was 100%. At 70 °C for 5 h, 85% of original PG activity was retained. A residual activity of 56% was noted even after 5 h of incubation at 80 °C (Figure 5B). A high residual activity of 45% and 43% at 90 °C after 4 and 5 h of incubation, respectively, indicated the potentiality of the thermostable enzyme. The PG reported in this research seems to be different from formerly reported enzymes in terms of adaptability for temperature and pH.

### 3.8. Mode of Action of PG

**Figure 6:**
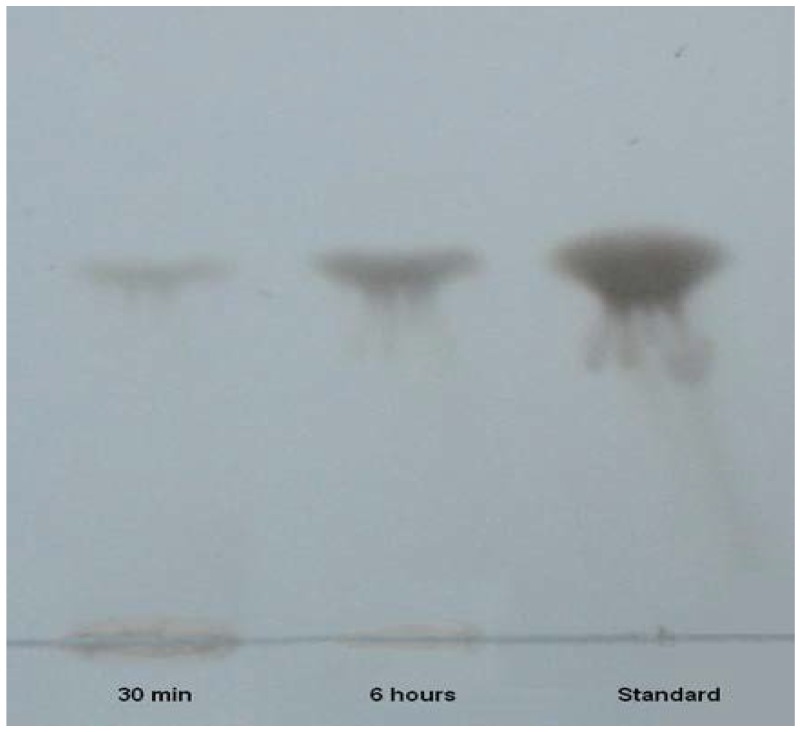
Thin layer chromatography of the reaction products of polygalacturonic acid hydrolyzed by PG. Hydrolysis times were 30 min and 6 h. Standard was 10 g/L of monogalacturonic acid.

Thin layer chromatography of hydrolysis products of PG from polygalacturonic acid after a reaction period of 30 min and 6 h, revealed the presence of one spot which corresponded to the standard monogalacturonic acid (Figure 6). PG appeared to be an exo-polygalacturonase acting at the end of the polygalacturonic chain.

### 3.9. Effect of the PG on the Quality of Apple and Citrus Juice

**Figure 7:**
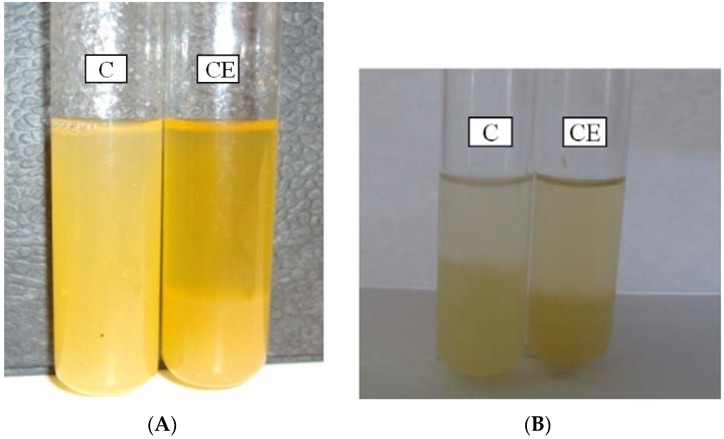
(**A**) Reduction of the haze of fresh citrus juice by CE (crude extracellular enzymes from *A. pullulans*); C: without treatment (considered as “control”); (**B**) Reduction of the haze of fresh apple juice by CE (crude extracellular enzymes from *A. pullulans*); C: without treatment (considered as “control”).

The capacity of exo-PG to degrade insoluble pectin molecules into soluble galacturonic acid was tested on a freshly extracted apple and citrus juice. Five mL of juice were incubated with crude enzymes (CE) for 4 h at 30 °C. The treated and non-treated samples were then left to decant for 48 h at 4 °C and photographed (Figure 7A,B). A clear net difference was observed between the treated and control tube. The analysis of the clarified juice showed a noticeable increase in terms of supernatant, at 30% (= (4 mL − 2.5 mL)/5 mL) and 60% (= (4 mL − 1mL)/5 mL), using CE to clarify citrus and apple juice, respectively, as compared to the non-treated sample, considered as control (Table 8A,B). The crude enzyme provided 30% and 60% of clarification of citrus and apple juices. Table 8A,B also shows that the amount of reducing sugars released after enzyme treatment in the apple and citrus juice was highly increased while the dry weight and A_420_ decreased.

## 4. Conclusions

Among many available methods for improving enzyme production, statistical optimization of media components using RMS continues to be a feasible and facile approach. In the present study, the optimization of variables (final composition: 40 g/L tomato pomace enriched by 1.84 g/L of lactose and 0.089 g/L of calcium chloride at pH 5.16) induced a 5-fold increase of the PG activity compared to that observed under unoptimized conditions. To our knowledge, this was the first study reporting the use of experimental designs to optimize PG production using the *A. pullulans* strain (isolated from a Saharan soil sample) grown on by-products of tomato manufacturing (tomato pomace) as a basal medium. Therefore, the results herein reported could be worthwhile for a possible future industrial application and for solving the waste disposal problem of tomato processing manufacturers. The crude enzymatic extract showed a high optimum temperature (60 °C) with good thermostability. In terms of pH stability, the PG from *A. pullulans* was an exo-PG and had activity and stability over a wide pH range. The PG could be commercialized for different industrial processes after purification. The enzyme presented in this work was applied successfully for the apple and citrus juice clarification. It also can be considered as a potential candidate in food industry.

In conclusion, this study points out that tomato pomace could be used as a promising industrial resource for PG production using the yeasts. In fact, such cheap waste streams, produced every year in huge amounts, could contribute positively to the implementation of viable cyclic economy utilizing pectins as a valuable component of enzyme production.

## Figures and Tables

**Table 1:** Range of different variables studied in the Plackett–Burman design (PBD).

Variables	Variable Code	Low Level (−1)	Low Level (+1)
pH	X_1_	5	6
Lactose	X_2_	0%	1%
Error	X_3_	-	-
Glucose	X_4_	0%	1%
Error	X_5_	-	-
CaCl_2_	X_6_	0 g/L	0.125 g/L
Yeast extract	X_7_	0%	0.2%

**Table 2:** Plackett-Burman design (PBD) of variables (in coded levels) with polygalacturonase (PG) activity as response.

Run Order	Experimental Values							PG Activity (U/mL)
	X_1_	X_2_	X_3_	X_4_	X_5_	X_6_	X_7_	
1	+1	+1	+1	−1	+1	−1	−1	7.67 ± 0.12
2	+1	+1	−1	+1	−1	−1	+1	7.82 ± 0.09
3	+1	−1	+1	−1	−1	+1	+1	3.84 ± 0.15
4	−1	+1	−1	−1	+1	+1	+1	4.55 ± 0.11
5	+1	−1	−1	+1	+1	+1	−1	4.08 ± 0.10
6	−1	−1	+1	+1	+1	−1	+1	4.13 ± 0.14
7	−1	+1	+1	+1	−1	+1	−1	5.61 ± 0.12
8	−1	−1	−1	−1	−1	−1	−1	4.13 ± 0.12

**Table 3:** Experimental codes, ranges and levels of the independent variables for response surface methodological experiments.

Variables	Symbols	Coded Levels				
		−α (−1.68)	−1	0	+1	+α (+1.68)
pH	X_1_	5.16	5.5	6	6.5	6.84
Lactose (%)	X_2_	0.16	0.5	1	1.5	1.84
CaCl_2_ (g/L)	X_3_	0.0205	0.0375	0.0625	0.0875	0.104

**Table 4:** Effects of the variables and statistical analysis of the Plackett-Burman design (PBD).

Variables	Effect	Coef.	SE Coef.	*t*-Value	*p*-Value	Confidence Level (%)
Constant		5.2275	0.1044	50.05	0.000	‒
pH	1.2490	0.6245	0.1044	5.98	0.027 ^b^	97.3
Lactose	2.3700	1.1850	0.1044	11.53	0.008 ^b^	99.2
Glucose	0.3620	0.1810	0.1044	1.73	0.225 ^a^	77.5
CaCl_2_	−1.4160	−0.7080	0.1044	−6.78	0.021 ^c^	97.9
Yeast extract	−0.2870	−0.1435	0.1044	−1.37	0.303 ^a^	69.7

Predicted-*R*^2^ = 0.8527; Adjusted-*R*^2^ = 0.9908. Coef.: coefficient; SE coef.: standard error coefficient; *t-*value: Student’s test value; *p-*value: probability value; ^a^ Non-significant at *p* < 0.05; ^b^ Significant positive effect; ^c^ Significant negative effect.

**Table 5:** Central composite design for the experimental and the corresponding responses to potentiation of PG activity.

Run Order	Coded Levels			Y Response (PG Activity) (U/mL)	
	X_1_	X_2_	X_3_	Experimental Value	Predicted Value
1	−1	−1	−1	10.76 ± 0.09	11.18
2	1	−1	−1	10.42 ± 0.07	10.16
3	−1	1	−1	14.16 ± 0.03	15.16
4	1	1	−1	11.50 ± 0.03	11.62
5	−1	−1	1	10.42 ± 0.10	10.62
6	1	−1	1	11.50 ± 0.07	10.81
7	−1	1	1	17.96 ± 0.11	18.53
8	1	1	1	16.31 ± 0.06	16.21
9	–1.682	0	0	17.35 ± 0.06	15.77
10	1.682	0	0	12.16 ± 0.09	12.96
11	0	–1.682	0	9.20 ± 0.10	9.54
12	0	1.682	0	18.22 ± 0.11	17.42
13	0	0	–1.682	10.42 ± 0.03	9.80
14	0	0	1.682	13.03 ± 0.09	13.18
15	0	0	0	14.27 ± 0.06	15.16
16	0	0	0	14.97 ± 0.06	14.31
17	0	0	0	14.16 ± 0.06	14.31

**Table 6:** Analysis of variance (ANOVA) for the parameters of response surface methodology fitted to quadratic polynomial model for optimization of potentiation of PG activity.

Source	SS	DF	MS	*F*-Value	*p*-Value
Model	124.887	9	13.8763	12.66	0.001
Residual error	7.674	7	1.0963		
Lackoffit	7.346	6	1.2244	3.73	0.377
Pure error	0.328	1	0.3281		
Total	132.561	16			

*R*^2^ = 94.21%; Adjusted-*R*^2^ = 86.77%; Predicted-*R*^2^ = 85.58%; SS, sum of squares; DF, degree of freedom; MS, mean square; *R*^2^: coefficient of determination.

**Table 7:** Results of regression analysis of the second-order polynomial model for optimization of potentiation of PG activity.

Factors	Coefficient	Estimated Coefficient	*t-*Value	*p*-Value
Constant	14.315	0.676	27.17	0.000
X_1_	−1.402	0.463	−3.03	0.019
X_2_	3.938	0.476	8.27	0.000
X_3_	1.691	0.476	3.55	0.009
X_1_^2^	0.052	0.959	0.05	0.958
X_2_^2^	−0.834	0.919	−0.91	0.394
X_3_^2^	−2.819	0.919	−3.07	0.018
X_1_X_2_	−1.78	1.04	−1.71	0.132
X_1_X_3_	0.86	1.04	0.82	0.439
X_2_X_3_	2.78	1.04	2.66	0.033

**Table 8:** (**A**) Effect of crude enzyme on the clarification rate of citrus juice. The reducing sugars, the volume and A_420_ were measured in the supernatant and the dry weight in the pellet, after centrifugation of treated citrus juice; (**B**) Effect of crude enzyme on the clarification rate of apple juice. The reducing sugars, the volume and A_420_ were measured in the supernatant and the dry weight in the pellet, after centrifugation of treated apple juice.

(**A**)		
	**Control**	**+ Crude Enzymes**
Reducing sugars (mg/mL)	4.7 ± 0.010	11.22 ± 0.049
Volume of supernatant (mL)	2.5 ± 0.000	4.000 ± 0.000
Dry weight (g)	1.250 ± 0.003	0.150 ± 0.045
A_420_	1.6 ± 0.002	0.739 ± 0.01
(**B**)		
	**Control**	**+ Crude Enzymes**
Reducing sugars (mg/mL)	95.07 ± 0.115	115.00 ± 0.045
Volume of supernatant (mL)	1.0 ± 0.000	4.000 ± 0.000
Dry weight (g)	2.0 ± 0.055	0.126 ± 0.008
A_420_	1.8 ± 0.520	0.553 ± 0.111

## References

[1] Food and Agriculture Organization of the United Nations (FAO) FAOSTAT, Production. http://faostat.fao.org/site/567/default.aspx#ancor.

[2] Al-Wandawi H., Abdul-Rahman M., Al-Shaikhly K. (1985). Tomato processing wastes as essential raw materials source. J. Agric. Food Chem..

[3] Strati I.F., Oreopoulou V. (2014). Recovery of carotenoids from tomato processing by-products—A review. Food Res. Int..

[4] Knoblich M., Anderson B., Latshaw D. (2005). Analyses of tomato peel and seed byproducts and their use as a source of carotenoids. J. Sci. Food Agric..

[5] Al-Muhtaseb A.A.H., Al-Harahsheh M., Hararah M., Magee T.R.A. (2010). Drying characteristics and quality change of unutilized-protein rich-tomato pomace with and without osmotic pre-treatment. Ind. Crops Prod..

[6] Del Valle M., Cámara M., Torija M.-E. (2006). Chemical characterization of tomato pomace. J. Sci. Food Agric..

[7] Grassino A.N., Halambek J., Djaković S., Rimac Brnčić S., Dent M., Grabarić Z. (2016). Utilization of tomato peel waste from canning factory as a potential source for pectin production and application as tin corrosion inhibitor. Food Hydrocoll..

[8] Pinela J., Barros L., Carvalho A.M., Ferreira I.C.F.R. (2012). Nutritional composition and antioxidant activity of four tomato (*Lycopersicon esculentum* L.) farmer’ varieties in Northeastern Portugal homegardens. Food Chem. Toxicol..

[9] Blandino A., Iqbalsyah T., Pandiella S., Cantero D., Webb C. (2002). Polygalacturonase production by *Aspergillus awamori* on wheat in solid-state fermentation. Appl. Microbiol. Biotechnol..

[10] Patil S.R., Dayanand A. (2006). Production of pectinase from deseeded sunflower head by *Aspergillus niger* in submerged and solid-state conditions. Bioresour. Technol..

[11] Vendruscolo F., Albuquerque P.M., Streit F., Esposito E., Ninow J.L. (2008). Apple pomace: A versatile substrate for biotechnological applications. Crit. Rev. Biotechnol..

[12] Buyukkileci A.O., Lahore M.F., Tari C. (2015). Utilization of orange peel, a food industrial waste, in the production of exo-polygalacturonase by pellet forming *Aspergillus sojae*. Bioprocess Biosyst. Eng..

[13] Díaz A.B., Alvarado O., de Ory I., Caro I., Blandino A. (2013). Valorization of grape pomace and orange peels: Improved production of hydrolytic enzymes for the clarification of orange juice. Food Bioprod. Process..

[14] Willats W.G.T., Knox J.P., Mikkelsen J.D. (2006). Pectin: New insights into an old polymer are starting to gel. Trends Food Sci. Technol..

[15] Demir H., Tarı C. (2014). Valorization of wheat bran for the production of polygalacturonase in SSF of *Aspergillus sojae*. Ind. Crops Prod..

[16] Jayani R.S., Saxena S., Gupta R. (2005). Microbial pectinolytic enzymes: A review. Process Biochem..

[17] Kashyap D.R., Vohra P.K., Chopra S., Tewari R. (2001). Applications of pectinases in the commercial sector: A review. Bioresour. Technol..

[18] Freedonia Group Inc. (2008). Enzymes, US Industry Study with Forecasts for 2012 & 2017.

[19] Taskin M. (2013). Co-production of tannase and pectinase by free and immobilized cells of the yeast *Rhodotorula glutinis* MP–10 isolated from tannin-rich persimmon (*Diospyros kaki* L.) fruits. Bioprocess Biosyst. Eng..

[20] Da Silva E.G., Borges M.D.F., Medina C., Piccoli R.H., Schwan R.F. (2005). Pectinolytic enzymes secreted by yeasts from tropical fruits. FEMS Yeast Res..

[21] Rashad M.M., Abdou H.M., Shousha W.G., Ali M.M., El-Sayed N.N. (2011). Purification and characterization of the pectin lyase produced by *Pleurotus ostreatus* grown on lemon pulp waste. Aust. J. Basic Appl. Sci..

[22] Arcuri S.L., Pagnocca F.C., da Paixão Melo W.G., Nagamoto N.S., Komura D.L., Rodrigues A. (2014). Yeasts found on an ephemeral reproductive caste of the leaf-cutting ant *Atta sexdens rubropilosa*. Antonie van Leeuwenhoek.

[23] Galiotou-Panayotou M., Kalantzi O., Aggelis G. (1998). Modelling of simultaneous production of polygalacturonase and exopolysaccharide by *Aureobasidium pullulans* ATHUM 2915. Antonie van Leeuwenhoek.

[24] Merín M.G., Mendoza L.M., Farías M.E., Morata de Ambrosini V.I. (2011). Isolation and selection of yeasts from wine grape ecosystem secreting cold-active pectinolytic activity. Int. J. Food Microbiol..

[25] Molnárová J., Vadkertiová R., Stratilová E. (2014). Extracellular enzymatic activities and physiological profiles of yeasts colonizing fruit trees. J. Basic Microbiol..

[26] Oliveira R.Q., Rosa C.A., Uetanabaro A.P.T., Azeredo A., Neto A.G., Assis S.A. (2009). Polygalacturonase secreted by yeasts from Brazilian semi-arid environments. Int. J. Food Sci. Nutr..

[27] Stratilová E., Dzúrová M., Breierová E., Omelková J. (2005). Purification and Biochemical Characterization of Polygalacturonases Produced by *Aureobasidium pullulans*. Z. Naturforschung C.

[28] Montgomery D. (2000). The present state of industrial statistics. Qual. Reliab. Eng. Int..

[29] Singh A.K., Mehta G., Chhatpar H.S. (2009). Optimization of medium constituents for improved chitinase production by *Paenibacillus sp.* D1 using statistical approach. Lett. Appl. Microbiol..

[30] Liu C., Liu Y., Liao W., Wen Z., Chen S. (2003). Application of statistically-based experimental designs for the optimization of nisin production from whey. Biotechnol. Lett..

[31] Belmessikh A., Boukhalfa H., Mechakra-Maza A., Gheribi-Aoulmi Z., Amrane A. (2013). Statistical optimization of culture medium for neutral protease production by *Aspergillus oryzae*. Comparative study between solid and submerged fermentations on tomato pomace. J. Taiwan Inst. Chem. Eng..

[32] Bennamoun L., Meraihi Z., Dakhmouche S. (2004). Utilisation de la planification expérimentale pour l’optimisation de la production de l’α-amylase par *Aspergillus oryzae* Ahlburg (Cohen) 1042.72 cultivé sur milieu à base de déchets d’oranges. J. Food Eng..

[33] Jung D.U., Yoo H.Y., Kim S.B., Lee J.H., Park C., Kim S.W. (2015). Optimization of medium composition for enhanced cellulase production by mutant *Penicillium brasilianum* KUEB15 using statistical method. J. Ind. Eng. Chem..

[34] Papagora C., Roukas T., Kotzekidou P. (2013). Optimization of extracellular lipase production by *Debaryomyces hansenii* isolates from dry-salted olives using response surface methodology. Food Bioprod. Process..

[35] Uzuner S., Cekmecelioglu D. (2015). Enhanced pectinase production by optimizing fermentation conditions of *Bacillus subtilis* growing on hazelnut shell hydrolyzate. J. Mol. Catal. B Enzym..

[36] Toksoy Öner E. (2006). Optimization of ethanol production from starch by an amylolytic nuclear petite *Saccharomyces cerevisiae* strain. Yeast.

[37] Bulatović M.L., Rakin M.B., Vukašinović-Sekulić M.S., Mojović L.V., Krunić T.Ž. (2014). Effect of nutrient supplements on growth and viability of *Lactobacillus johnsonii* NRRL B–2178 in whey. Int. Dairy J..

[38] Yu X., Hallett G.S., Sheppard J., Watson K.A. (1997). Application of the Plackett-Burman experimental design to evaluate nutritional requirements for the production of *Colletotrichum coccodes* spores. Appl. Microbiol. Biotechnol..

[39] Buzzini P., Martini A. (2002). Extracellular enzymatic activity profiles in yeast and yeast-like strains isolated from tropical environments. J. Appl. Microbiol..

[40] Kurtzman C.P., Fell J.W., Boekhout T., Robert V. (2011). Methods for Isolation, Phenotypic Characterization and Maintenance of Yeasts. The Yeasts.

[41] Sampaio J.P., Gadanho M., Santos S., Duarte F.L., Pais C., Fonseca A., Fell J.W. (2001). Polyphasic taxonomy of the basidiomycetous yeast genus *Rhodosporidium*: *Rhodosporidium kratochvilovae* and related anamorphic species. Int. J. Syst.Evolut. Microbiol..

[42] Basic Local Alignment Search Tool. https://blast.ncbi.nlm.nih.gov/Blast.cgi.

[43] Dereeper A., Guignon V., Blanc G., Audic S., Buffet S., Chevenet F., Dufayard J.-F., Guindon S., Lefort V., Lescot M. (2008). Phylogeny.fr: Robust phylogenetic analysis for the non-specialist. Nucleic Acids Res..

[44] DBVPG—Industrial Yeasts Collection. http://www.dbvpg.unipg.it.

[45] Miller G.L. (1959). Use of dinitrosalicylic acid reagent for determination of reducing sugar. Anal. Chem..

[46] Plackett R.L., Burman J.P. (1946). The Design of Optimum Multifactorial Experiments. Biometrika.

[47] Box G.E.P., Wilson K.B. (1959). On the experimental attainment of optimum conditions. J.R. Stat. Soc. B.

[48] Contreras Esquivel J.C., Voget C.E. (2004). Purification and partial characterization of an acidic polygalacturonase from *Aspergillus kawachii*. J. Biotechnol..

[49] Bhardwaj V., Garg N. (2014). Pectinase production by *Delftia Acidovorans* isolated from fruit waste under submerged fermentation. Int. J.Sci. Res..

[50] Malvessi E., Silveira M.M.d. (2004). Influence of medium composition and pH on the production of polygalacturonases by *Aspergillus oryzae*. Braz. Arch. Biol. Technol..

[51] Martínez-Trujillo A., Aranda J.S., Gómez-Sánchez C., Trejo-Aguilar B., Aguilar-Osorio G. (2009). Constitutive and inducible pectinolytic enzymes from *Aspergillus flavipes* FP–500 and their modulation by pH and carbon source. Braz. J. Microbiol..

[52] Oncu S., Tari C., Unluturk S. (2007). Effect of various process parameters on morphology, rheology, and polygalacturonase production by *Aspergillus sojae* in a batch bioreactor. Biotechnol. Prog..

[53] Suresh B., Viruthagiri T., Sasikumar E. (2009). Optimization of process variables using Response Surface Methodology (RSM) in the solid-state fermentative production of pectinase by *Aspergillus awamori*. Asian J. Food Agro-Ind..

[54] Mukesh Kumar D.J., Saranya G.M., Suresh K., Andal Priyadharshini D., Rajakumar R., Kalaichelvan P. (2012). Production and Optimization of Pectinase from *Bacillus sp.* MFW7 using Cassava Waste. Asian J. Plant Sci. Res..

[55] Chaudhri A., Suneetha V. (2012). Microbially derived pectinases: A review. IOSR J Pharm Biol Sci..

[56] Gomes E., Aguiar A.P., Carvalho C.C., Bonfá M.R.B., Silva R.d., Boscolo M. (2009). Ligninases production by *Basidiomycetes* strains on lignocellulosic agricultural residues and their application in the decolorization of synthetic dyes. Braz. J. Microbiol..

[57] Murthy P.S., Naidu M.M. (2010). Protease production by *Aspergillus oryzae* in solid-state fermentation utilizing coffe by-pro-ducts. World Appl. Sci. J..

[58] Moyo S., Gashe B.A., Collison E.K., Mpuchane S. (2003). Optimising growth conditions for the pectinolytic activity of *Kluyveromyces wickerhamii* by using response surface methodology. Int. J. Food Microbiol..

[59] Whitaker J.R., Fogarty W.M., Kelly C.T. (1990). Microbial Pectolytic Enzymes. Microbial Enzymes and Biotechnology.

[60] Kashyap D.R., Chandra S., Kaul A., Tewari R. (2000). Production, purification and characterization of pectinase from a *Bacillus sp.* DT7. World J. Microbiol. Biotechnol..

[61] Umsza-Guez M.A., Díaz A.B., Ory I.D., Blandino A., Gomes E., Caro I. (2011). Xylanase production by *Aspergillus awamori* under solid state fermentation conditions on tomato pomace. Braz. J. Microbiol.S.

[62] Sampaio P.N., Calado C.R.C., Sousa L., Bressler D.C., Pais M.S., Fonseca L.P. (2010). Optimization of the culture medium composition using response surface methodology for new recombinant cyprosin B production in bioreactor for cheese production. Eur. Food Res. Technol..

[63] Shanmugaprakash M., Kirthika J., Ragupathy J., Nilanee K., Manickam A. (2014). Statistical based media optimization and production of naringinase using *Aspergillus brasiliensis* 1344. Int. J. Biol. Macromol..

[64] do Rosário Freixo M., Karmali A., Arteiro J.M. (2008). Production of polygalacturonase from *Coriolus versicolor* grown on tomato pomace and its chromatographic behaviour on immobilized metal chelates. J. Ind. Microbiol. Biotechnol..

[65] Sharma D.C., Satyanarayana T. (2006). A marked enhancement in the production of a highly alkaline and thermostable pectinase by *Bacillus pumilus* DCSR1 in submerged fermentation by using statistical methods. Bioresour. Technol..

[66] Tari C., Gögus N., Tokatli F. (2007). Optimization of biomass, pellet size and polygalacturonase production by *Aspergillus sojae* ATCC 20235 using response surface methodology. Enzym. Microb. Technol..

[67] Niture S.K. (2008). Comparative biochemical and structural characterizations of fungal polygalacturonases. Biologia.

[68] Yuan P., Meng K., Wang Y., Luo H., Shi P., Huang H., Bai Y., Yang P., Yao B. (2012). A protease-resistant exo-polygalacturonase from *Klebsiella* sp. Y1 with good activity and stability over a wide pH range in the digestive tract. Bioresour. Technol..

[69] Manachini P.L., Parini C., Fortina M.G. (1988). Pectic enzymes from *Aureobasidium pullulans* LV 10. Enzym. Microb. Technol..

